# Molecular Insights into Lignin Bioactivity: From Structural Architecture to Sustainable Food Industry Applications

**DOI:** 10.3390/ijms27104458

**Published:** 2026-05-15

**Authors:** Akhmadjon Sultanov, Rakhmat Sultonov, Byung-Dae Park, Ju-Ock Nam, Soo Rin Kim, Deokyeol Jeong

**Affiliations:** 1 School of Food Science and Biotechnology, Kyungpook National University, Daegu 41566, Republic of Korea; ahmatjon.net@gmail.com (A.S.); namjo@knu.ac.kr (J.-O.N.); 2 Department of Chemistry, Kyungpook National University, Daegu 41566, Republic of Korea; sultonov.rakhmat@knu.ac.kr; 3 Department of Analytical Chemistry, Samarkand State University, Samarkand 140104, Uzbekistan; 4 Department of Wood and Paper Science, Kyungpook National University, Daegu 41566, Republic of Korea; byungdae@knu.ac.kr; 5 Research Institute of Tailored Food Technology, Kyungpook National University, Daegu 41566, Republic of Korea; 6 Department of Food Science and Technology, Kongju National University, Yesan 32439, Republic of Korea

**Keywords:** lignin, antioxidant, antimicrobial, anti-inflammatory, functional foods

## Abstract

This review explores the biological properties and application potential of native, technical, and modified lignins, with a focus on their antioxidant, antimicrobial, and anti-inflammatory activities. Native lignin generally preserves more of its original phenolic architecture and thus shows stronger intrinsic biological activity. This is likely due to its more homogeneous structure, which makes its physicochemical behavior more predictable compared with highly processed technical lignins. Among technical lignins, organosolv and soda lignin appear the most promising due to their sulfur-free nature, lower condensation, and higher reactivity. At the monomer level, catechol-type phenolics show the highest antioxidant potential, while vanillin remains the most attractive lignin-derived monomer because it combines bioactivity with direct application potential in food, pharmaceutical, and cosmetic systems. Comparison of modification strategies indicates that phenolic grafting, esterification, and carboxylation are more practical for scale-up than complex multistep polymer grafting. In particular, gallic acid grafting produced some of the strongest results, including near-complete 2,2′-azino-bis-(3-ethylbenzothiazoline-6-sulfonic acid) (ABTS) scavenging, 98.7% 2,2-diphenyl-1-picrylhydrazyl (DPPH) radical inhibition, and a fourfold increase in phenolic hydroxyl content, whereas other modified lignins also showed improved antimicrobial and anti-inflammatory effects. Overall, mild and green lignin modification, especially with food-safe phenolic compounds, appears to be the most promising strategy for future food and human health applications.

## 1. Introduction

Lignin is a plant cell-wall polyphenolic polymer deposited mainly in secondary walls, where it reinforces the lignocellulosic matrix and contributes to water resistance and defense against microbial attack [1]. Structurally, lignin is not a uniform linear biopolymer but an irregular, three-dimensionally crosslinked network formed by oxidative radical coupling of monolignols such as *p*-coumaryl, coniferyl, and sinapyl alcohols. This process generates *p*-hydroxyphenyl (H), guaiacyl (G), and syringyl (S) units with diverse C-O and C-C interunit linkages [1,2]. This inherent structural complexity contributes to longstanding analytical challenges and makes lignin difficult to standardize as an engineering feedstock [1,3].

Despite its abundance, lignin remains underutilized as a value-added resource. While very large quantities of technical lignin are generated as coproducts of pulping and biorefinery operations, much of this material is still combusted for on-site energy rather than upgraded into higher value aromatics or functional materials [2,4]. Crucially, technical lignins are not interchangeable, including kraft, sulfite, soda, and organosolv processes, which impose distinct chemical transformations in fragmentation, condensation, and sulfonation. These processes produce different molar-mass distributions and functional group patterns that govern solubility, reactivity, and downstream performance [3]. Functionality can also vary across the molar-mass distribution, so bulk-average descriptors may miss the fractions most responsible for bioactivity or interfacial behavior [2,5].

The increasing interest in lignin for biological and food-relevant functions follows directly from its phenolic chemistry. Phenolic hydroxyl groups can quench radicals via hydrogen-atom or single-electron transfer mechanisms and form stabilized phenoxy radicals. In addition, aromatic chromophores absorb UV radiation, a feature relevant to limiting lipid oxidation, photooxidation, and microbially driven spoilage or quality loss [6,7,8]. Food-relevant lignin chemistry also appears at the monomer level. Oxidative depolymerization can yield aromatic aldehydes and acids such as vanillin and syringaldehyde, with yields and selectivity strongly dependent on lignin source, extraction severity, and reaction conditions [4,9]. Vanillin is among the most widely used flavoring agents worldwide and is extensively employed in food. Although petrochemical synthesis dominates global supply, lignin-derived routes represent an established industrial pathway that is still practiced commercially [5,9,10]. Lignin-derived low molecular weight (M_w_) phenolics can also show express antioxidant or antimicrobial effects, motivating lignin modification pathways aligned with food-relevant functionalities rather than fuel-only use [4,11,12].

In the food sector, lignin and grafted or modified lignin are currently most mature as functional components in food contact materials, coatings, and interfacial delivery systems rather than as direct ingestible additives [8]. Because dispersion and compatibility frequently limit performance, chemical modification and grafting are increasingly used as design tools. For example, polylactic acid (PLA)-grafted lignin nanoparticles were shown to disperse more uniformly in PLA and to enhance antioxidant and UV-barrier performance relative to unmodified lignin fillers [13]. Etherification has similarly been used to tune lignin–PLA compatibility, with studies that explicitly report migration testing illustrating how modification choices intersect with food-contact requirements. Lignin particles can also act as Pickering emulsifiers in lignin nanoparticle-stabilized clove essential oil emulsions. These emulsions have been incorporated into starch films for cherry and tomato preservation, combining sustained release with antioxidant and antibacterial effects [14]. Finally, grafted lignin copolymers have been designed to stabilize bioactive loaded oil-in-water systems and enable stimulus responsive release, exemplified by lignin-graft-PNIPAM nanoparticles stabilizing trans resveratrol in palm oil emulsions [4,15].

Many reviews already catalog lignin modification routes and lignin-enabled materials across sectors, including food-related materials and packaging [2,6,8,12]. Yet, for food applications, the evidence base remains difficult to synthesize critically because structure–activity relationships are often asserted but are not built from comparable datasets. Cross-study comparability is weakened by inconsistent reporting of lignin provenance, industrial process, purification and fractionation method, and modification or grafting chemistry [3,4,5,16]. Outcomes are further confounded by non-uniform structural characterization, including molar-mass distribution, OH-group quantification and S/G/H composition despite evidence that both functional groups and heterogeneity can drive performance [5,6,7]. Finally, studies employ different antioxidant and antimicrobial assays and reporting units. This variation can obscure whether observed improvements reflect intrinsic chemical reactivity or improved dispersion and mass transfer in a given test system [6,7,9].

The objective of this review is therefore to assess lignin and chemically modified or grafted lignin as sustainable bio-based alternatives for food industry applications through a structure–activity lens. We focus on how lignin source, molecular structure, and intentional chemical modification including grafting and nanoparticle engineering influence antioxidant, antimicrobial, UV-barrier, and interfacial functions in food systems and food packaging.

## 2. Types and Sources of Lignin

Historically relegated to a low-value, recalcitrant by-product of the global pulp and paper industry, lignin is increasingly recognized as the most abundant renewable source of aromatic carbon on earth [17]. However, the transition of lignin from a combustible waste stream to a high-value biorefinery feedstock is severely hampered by its inherent structural heterogeneity and the extensive chemical degradation it suffers during industrial extraction [18,19]. A critical evaluation of lignin must distinguish between its native botanical architecture and the heavily modified technical variants generated by industrial processing (Table 1).

### 2.1. Lignin Biosynthesis and Lignification

**Figure 1:**
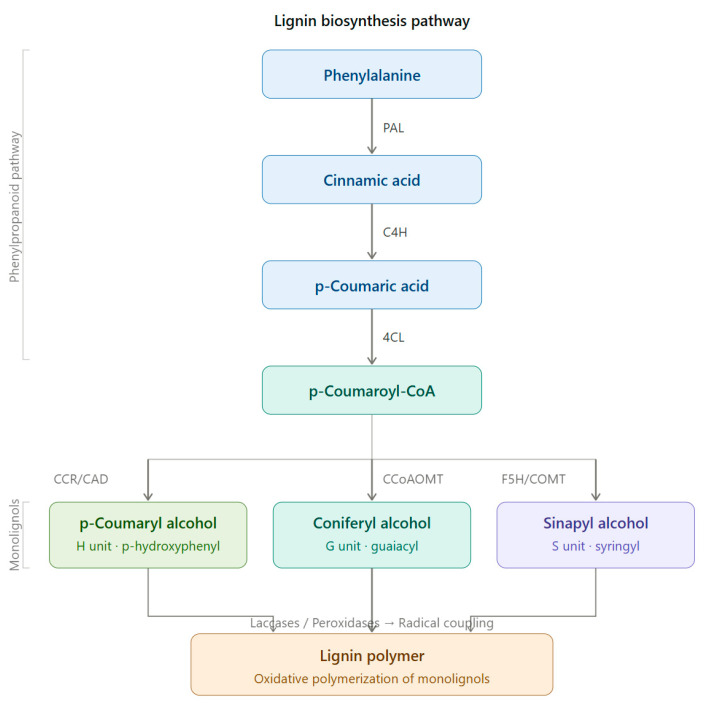
Phenylpropanoid pathway in lignin biosynthesis. PAL, phenylalanine ammonia-lyase; C4H, cinnamate 4-hydroxylase; 4CL, 4-coumarate-CoA ligase; CCR, cinnamoyl-CoA reductase; CAD, cinnamyl alcohol dehydrogenase; CCoAOMT, caffeoyl-CoA O-methyltransferase; F5H, ferulate 5-hydroxylase; COMT, caffeic acid O-methyltransferase.

Lignin is a biopolymer formed through monolignol biosynthesis, followed by transport to the cell wall, oxidation, and polymerization [1]. The monolignols of lignin consist of *p*-coumaryl alcohol (H), coniferyl alcohol (G), and sinapyl alcohol (S), which are biosynthesized via the phenylpropanoid pathway (Figure 1) using L-phenylalanine and tyrosine as precursors. This pathway includes hydroxylation and methylation steps involving enzymes such as hydroxycinnamoyl-CoA shikimate/Quinate hydroxycinnamoyltransferase (HCT), *p*-coumarate 3-hydroxylase (C3H), caffeoyl-CoA *O*-methyltransferase (CCoAOMT), ferulate 5-hydroxylase (F5H), and caffeic acid O-methyltransferase (COMT), which form various intermediates. It also includes reduction steps involving cinnamoyl-CoA reductase (CCR) and cinnamyl alcohol dehydrogenase (CAD), which convert these intermediates into monolignol alcohols [1,26]. Subsequently, monolignols synthesized in the cytoplasm are transported across the cell membrane into the apoplastic space through active transport, such as ATP-binding cassette transporter AtABCG29 or through passive diffusion. They are oxidized by laccases and peroxidases and converted into phenoxy radicals [1,26,27]. These radicals undergo combinatorial coupling reactions to form major inter-unit bonds such as β-O-4, β-5, 5-5, and β-β bonds, and the resulting polymerized lignins ultimately accumulate within the secondary cell wall as a heterogeneous three-dimensional aromatic network [1,26]. Importantly, lignin structure is determined not only by the supply of monolignols but also by the spatial distribution and substrate specificity of oxidases; these two factors jointly influence bonding patterns and polymer composition [1,27].

### 2.2. Native Botanical Lignin

The fundamental architecture of native lignin is inextricably linked to its botanical origin, which dictates its innate resistance to chemical and enzymatic deconstruction, commonly referred to as biomass recalcitrance. However, the composition of natural lignin in plants is not uniform and varies in various aspects, including the ratio of monolignols, the substitution patterns of aromatic rings, and the distribution of inter-unit bonds. These compositional differences form the basis for the distinct lignin structures observed in softwoods, hardwoods, and herbaceous plants [27].

Softwood lignin predominantly synthesizes G-units, leading to highly condensed matrices that contain a higher proportion of carbon–carbon (C-C) linkages, such as β-5 (phenylcoumaran) and 5-5′ (biphenyl) bonds, although β-O-4′ linkages typically remain the most abundant. This dense cross-linking makes softwood lignin difficult to delignify. In contrast, hardwood lignins are composed of both G- and S-units, with S-units being predominant. Because the S-unit is methoxylated at both the 3- and 5-positions, C-C coupling at the 5-position is sterically blocked, forcing the polymer to propagate via more labile β-O-4′ aryl ether bonds that often exceed 60–70% of total linkages [28,29]. Consequently, hardwood lignin is significantly more amenable to chemical depolymerization. Herbaceous crops further complicate this model by introducing a mixed H/G/S matrix interwoven with hydroxycinnamate bridges such as ferulate and *p*-coumarate, which critically dictate the digestibility of forage crops and the efficacy of agricultural biomass processing [26].

Crucially, the long-standing scientific consensus that lignification is a purely random, combinatorially assembled chemical process has faced intense critical scrutiny in recent years [30]. This debate was ignited by the discovery of catechyl lignin (C-lignin), a stereoregular, linear homopolymer of caffeyl alcohol (C_9_H_10_O_3_) found in the seed coats of plants such as *Vanilla planifolia* and various cacti. Composed almost entirely of 1,4-benzodioxane (C_8_H_8_O_2_) units and bypassing traditional G/S synthesis routes, C-lignin represents a profound departure from classical lignin models [31]. Its existence suggests that laccase specificity and spatial enzymatic control play a much more deterministic role in lignification than previously theorized [32]. From an industrial perspective, C-lignin’s linear, easily cleavable structure presents an ideal natural template for producing high-value catechol derivatives, illustrating how nature’s own variants might solve prevailing depolymerization challenges. Similarly, the recent confirmation that the flavonoid tricin acts as an authentic, integral monomer in monocot lignification shows that the polymer is a highly flexible, evolving sink for secondary metabolites rather than a rigid structural barrier [33].

### 2.3. Technical Lignin

In the context of industrial valorization, the botanical origin of lignin is often eclipsed by the severity of the extraction process. The “Carbohydrate First” dogma of the 20th-century pulping industry optimized cellulose recovery at the direct expense of lignin integrity [34]. The resulting technical lignins exhibit multiscale heterogeneity ranging from unpredictable M_w_ distributions to process-induced condensation, which severely impedes their use in high-performance polymeric materials [35].

#### 2.3.1. Kraft Lignin

Kraft pulping accounts for the vast majority of globally produced technical lignin [36]. The highly alkaline conditions, using NaOH and Na_2_S, actively cleave native ether bonds but simultaneously trigger profound secondary condensation [37]. Recent mechanistic studies have revealed that retro aldol reaction followed by radical recombination generates novel and highly recalcitrant C-C linkages. This condensation pathway, marked by diagnostic homovanillin-derived lactone species absent in native wood, creates a highly fragmented, cross-linked polymer. Compounded by the incorporation of 1–3% covalently bonded sulfur, kraft lignin is notoriously difficult to upgrade catalytically, effectively trapping its commercial utility in low-value thermal energy recovery or basic phenol-formaldehyde adhesive formulations [36].

#### 2.3.2. Lignosulfonates

Lignosulfonates, derived from acidic or neutral sulfite pulping, represent the largest commercialized fraction of functional lignin products. They are heavily sulfonated at the α-carbon, granting them exceptional water solubility and making them highly effective as concrete plasticizers, agricultural dispersants, and binders. However, this high degree of functionalization is a double-edged sword, since their massive, polydisperse M_w_ (up to 50,000 g/mol) and heavy sulfur contamination (5–8%) render them largely unsuitable for precision chemical depolymerization or integration into advanced carbon fibers [36].

#### 2.3.3. Organosolv Lignin and Soda Lignin

Seeking to overcome the limitations of sulfur-contaminated lignin, organosolv and soda extractions offer a more promising structural profile. Solvolysis in organic solvents such as ethanol or acetic acid selectively cleaves alkyl–aryl ether bonds without the extreme condensation seen in the kraft process, yielding low M_w_, highly pure, and sulfur-free oligomers [38]. Despite these superior physicochemical properties, which make them ideal precursors for polyurethane blends and specialty bioplastics, their industrial penetration remains limited. The prohibitive capital costs, high-pressure equipment requirements, and solvent recovery hurdles associated with scaling organosolv technologies continue to restrict this premium lignin to a niche market [35].

### 2.4. Emerging Solutions

The fundamental limitation of technical lignins is that they are downstream products of processes designed primarily to isolate cellulose. To circumvent the detrimental condensation reactions inherent to traditional biorefining, the field is currently undergoing a paradigm shift toward lignin-first extraction strategies. Beyond reductive catalytic fractionation, other emerging strategies include deep eutectic solvents (DESs), which enable mild, tunable dissolution of lignin with minimal structural modification and high β-O-4′ linkage preservation; ionic liquid-based extraction, which offers high selectivity and recyclability; and biotechnological approaches such as laccase- or peroxidase-assisted depolymerization, which can yield defined phenolic monomers under mild aqueous conditions.

These routes are included in Table 1 and represent complementary pathways toward high-quality, low-condensation lignin for bioactivity applications. This in situ passivation preserves a near-native abundance of aryl ether linkages and helps prevent the formation of intractable C-C condensation bonds. Although this approach successfully yields uniform, easily upgradable phenolic monomers, its commercial viability is currently constrained by complex chemical engineering challenges, primarily catalyst deactivation by biomass impurities and the energy-intensive nature of solvent recycling.

Nevertheless, the lignin-first approach represents a critical evolutionary step, shifting the narrative of lignin from a degraded, heterogeneous waste burden to a highly tailored, functional precursor essential for a sustainable circular bioeconomy.

## 3. Chemical Structure and Bioactivity of Lignin

Bioactivity claims for technical lignin are often unstable because lignin chemistry shifts during isolation and many assays are confounded by solubility, light absorption, and turbidity. The most consistent systemic acquired resistance (SAR) is that higher accessible phenolic hydroxyl content improves antioxidant and antimicrobial activity but can also increase cytotoxicity [39].

### 3.1. Lignin Monomers, Phenolics, and Structure Activity

**Figure 2:**
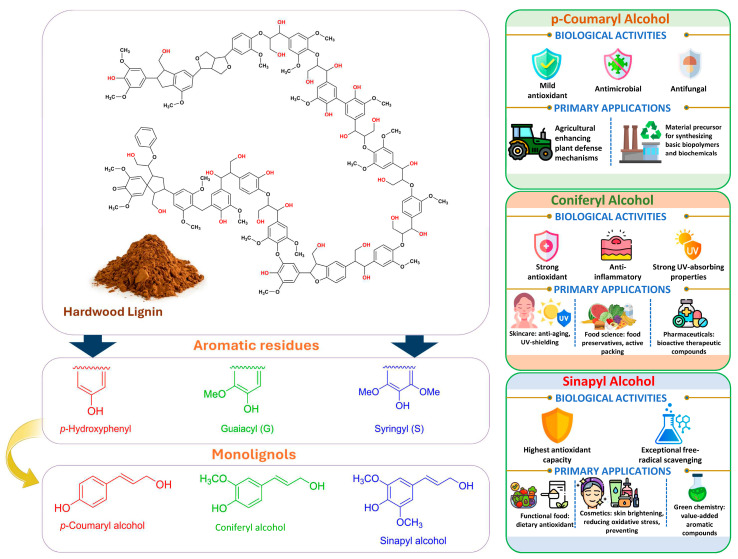
Biological properties and applications of lignin monolignols.

Technical lignin SAR depends on a coupled set of descriptors as follows: S/G/H ratio, β-O-4 content, condensed units (C–C), phenolic hydroxyls, methoxy groups, carboxyl groups, M_w_, and polydispersity index (PDI). β-O-4 cleavage during pulping and fractionation usually decreases M_w_ and creates new phenolic OH groups, strengthening H-donation/radical quenching antioxidant effects and, when soluble, membrane active antimicrobial effects [40]. Methoxy substitution plays a key role in stabilizing phenoxyl radicals. However, the correlation between S/G/H ratios and bioactivity is often inconsistent. This inconsistency arises because the S/G/H ratio closely co-varies with other structural parameters, including the degree of condensation, M_w_, PDI, and solubility. Consequently, factors such as condensed units and carboxylation can drastically shift the apparent activity depending on the testing environment, specifically whether the lignin is evaluated in a true solution, as a colloidal dispersion, or within a solid film (Figure 2) [7].

### 3.2. Biological Properties of Lignin

Antioxidant activity is well-documented but not standardized. 2,2-Diphenyl-1-picrylhydrazyl (DPPH) outcomes depend strongly on solvent, time to steady state, and lignin absorbance near 515 nm unless blanks and normalized indices are reported [41]. Antimicrobial activity is repeatedly observed across lignin types, but minimum inhibitory concentration (MIC) and minimum bactericidal concentration (MBC) values are poorly comparable across studies. Turbidity, precipitation, and inconsistent endpoints are common confounding factors that can dominate the apparent antimicrobial signal. Furthermore, the proposed mechanisms of action are frequently inferred from activity data rather than directly isolated through controlled experiments. Pro-inflammatory cytokine nuclear factor kappa-B (NF-κB)-associated and tyrosinase enzyme inhibition are reported, but they are often based on single model systems and may reflect non-specific polyphenol protein binding unless kinetics and impurity controls are applied, especially because the same low M_w_, phenolic-rich fractions can be broadly cytotoxic [42]. A key reproducibility gap is inconsistent reporting of phenolic-OH content by ^31^P nuclear magnetic resonance (NMR), linkages/condensation by heteronuclear single quantum coherence (HSQC), size-exclusion chromatography (SEC) M_w_/PDI, and ash/metal content [43].

Native and technical lignins, including kraft, organosolv, soda, and lignosulfonate lignins, differ in phenolic -OH content, methoxy density (G/S/H-unit ratio) (Table 2), condensation, M_w_, and charge. These factors shift both intrinsic activity and the effects of modifications. Softwood kraft fractions with higher phenolic -OH content tend to show stronger DPPH activity; lignin enriched in catechol-like units can outperform other lignins in radical consumption and show lower EC_50_ values in DPPH assays [44].

Mechanistically, lignin antioxidants primarily act via hydrogen-atom transfer (HAT), in which a phenolic O-H bond donates a hydrogen atom to a radical species (Ar-OH → Ar-O• + H•), generating a stabilized phenoxy radical. A secondary pathway is single-electron transfer (SET), in which an electron is transferred from the phenolic unit to the radical, followed by proton transfer, also yielding a resonance-stabilized phenoxy radical delocalized across the aromatic network [44,45]. Bioactivity in aqueous biological conditions is often dispersion-limited, as lignin with high intrinsic redox capacity can still be weak in practice if it aggregates and hides phenolic sites. Targeted functional group derivatization shows that blocking free phenolic OH groups markedly reduces measured antioxidant performance, demonstrating that phenolic accessibility is more diagnostically useful than phenolic OH content alone [44,45,46]. The biological activity of lignin varies significantly depending on its structure. Lignin derived from hardwood, which contains a higher proportion of S-units, tends to have a higher antioxidant activity than softwood lignin, which is more enriched in G-units. The presence of methoxy groups in S-units enhances radical scavenging properties, making hardwood lignin more effective in antioxidant applications [47,48]. For antimicrobial action, lignin and lignin particles can act through surface adhesion, membrane destabilization/permeabilization, metabolic suppression, and reactive oxygen species (ROS) elevation in bacteria. In a metal-free phenolated lignin nanoparticle system, increased ROS in bacteria and membrane disruption were directly linked to stronger antibacterial effects compared with nonfunctionalized lignin nanoparticles and phenolic lignin [49,50].

**Table 2:** Biological activities and potential applications of native and technical lignins.

Lignin Type	Bio-Applications	Antioxidant Activity (μg/mL, IC50)	Antimicrobial Activity (μg/mL, MIC)	Anti-Inflammatory (μg/mL, IC50)	Other Biological Activities	Key Notes	References
Ionic liquid lignin	Medical, Materials, Cosmetics, Biorefinery	50	100	75	Nanoparticle synthesis, drug encapsulation, antibiofilm, antitumor (in vitro), antifungal	High structural preservation; excellent reactivity; promising for advanced biomedical applications	[51,52,53,54]
Deep eutectic solvent lignin	Medical, Cosmetics, Biorefinery, Environment	45	120	80	Anticancer (apoptosis induction), anti-biofilm, antifungal, immunomodulatory, ROS scavenging	Highest bioactivity reported; green-solvent process; high β-O-4 linkage preservation; low toxicity	[55,56]
Kraft lignin	Medical, Materials, Biorefinery, Cosmetics	60	150	85	Anticancer, UV protection, drug delivery carrier, antiviral (HSV-1/2), wound healing	Most commercially available; sulfur-containing; high DPPH radical-scavenging activity	[55,57,58,59]
Lignosulphonates	Biorefinery, Agriculture, Materials, Environment	55	140	90	Dispersant, emulsifier, soil conditioner, heavy metal adsorption, antiviral (HIV, influenza)	Water-soluble; sulfonated groups enhance bioavailability; wide molecular weight distribution	[57,59]
Organosolv lignin	Medical, Cosmetics, Materials, Biorefinery	70	110	65	Anticancer, nanoparticle formation, UV-blocking, antifungal, antidiabetic (α-glucosidase inhibition)	Sulfur-free, high purity; excellent phenolic content; most favorable for pharma and cosmetic use	[36,57,60]
Soda lignin	Medical, Cosmetics, Agriculture, Biorefinery	80	160	100	Antiviral, anticancer cell-line inhibition, soil amendment, biostimulant in agriculture	Sulfur-free from non-wood biomass (wheat straw, flax); good for biomedical formulations	[51,58,59,61]
Steam explosion lignin	Biorefinery, Materials, Environment, Agriculture	90	170	110	Prebiotic/gut microbiome modulation, heavy metal chelation, antifungal, biocomposite filler	Partially depolymerized; increased surface area; moderate biological activity; lower purity	[57,62,63]
Pyrolysis lignin	Biorefinery, Materials, Environment	100	180	120	Biofuel additive, phenol precursor for resins, carbon fiber precursor, antifungal (low)	Depolymerized by thermal cleavage; complex low M_W_ phenolic mixture; limited biological use	[64,65]
Hydrolysis lignin	Biorefinery, Environment, Materials	110	190	130	Carbon material precursor, soil amendment, compost additive, heavy metal immobilization	Highly condensed post-acid hydrolysis; limited solubility; primarily used as fuel feedstock	[64,66]

## 4. Chemical Modification and Grafting Strategies of Lignin Bioactivity

Lignin’s antioxidant, antimicrobial, and anti-inflammatory functions arise primarily from (i) phenolic hydroxyl groups (H-donation/electron transfer), (ii) methoxy substitution patterns (radical stabilization), (iii) M_w_/condensation (phenolic accessibility), and (iv) supramolecular presentation (surface area, charge, and diffusivity). Recent work shows that chemical modification can either enhance bioactivity by increasing accessible phenolics and colloidal stability or suppress it by masking phenolic OH while improving compatibility with hydrophobic matrices. Examples that achieve large gains include carboxylation using maleic acid hydrotropic fractionation radical scavenging up to 98% DPPH and 94% 2,2′-azino-bis-(3-ethylbenzothiazoline-6-sulfonic acid) (ABTS) and gallic acid grafting near-complete ABTS neutralization and 98.7% DPPH scavenging, with a reported fourfold increase in phenolic OH [41,67]. For antimicrobial performance, cationization is the dominant lever. Quaternary ammonium lignin (QAL) can reach extremely low bactericidal concentrations for minimal bactericidal concentration as low as 0.012 mg/L against *Klebsiella pneumoniae* after 1 h, with strong dependence on alkyl chain length [68].

For anti-inflammatory activity, the strongest evidence in the last decade comes from the following two mechanistic classes: (i) polyanionic sulfonated lignin (lignosulfonates), which inhibits inflammation-related proteases and suppresses TNF-α to NF-κB signaling in cells, and (ii) engineered lignin graft copolymers/hydrogels, which down-regulate inflammatory mediators (iNOS, IL-1β) and attenuate NF-κB activation in macrophage models while simultaneously controlling infection [58,67]. The routes that best balance potency, scalability, and safety appear to be phenolic enrichment strategies (phenolation and gallic or tannic acid conjugation), ionic functionalization tailored to the biological target (sulfonation for anti-inflammatory bio-interfaces and quaternization for antimicrobials), and nano-formulation to increase phenolic exposure and microbial contact efficiency [41,49,68].

### 4.1. Esterification

**Figure 3:**
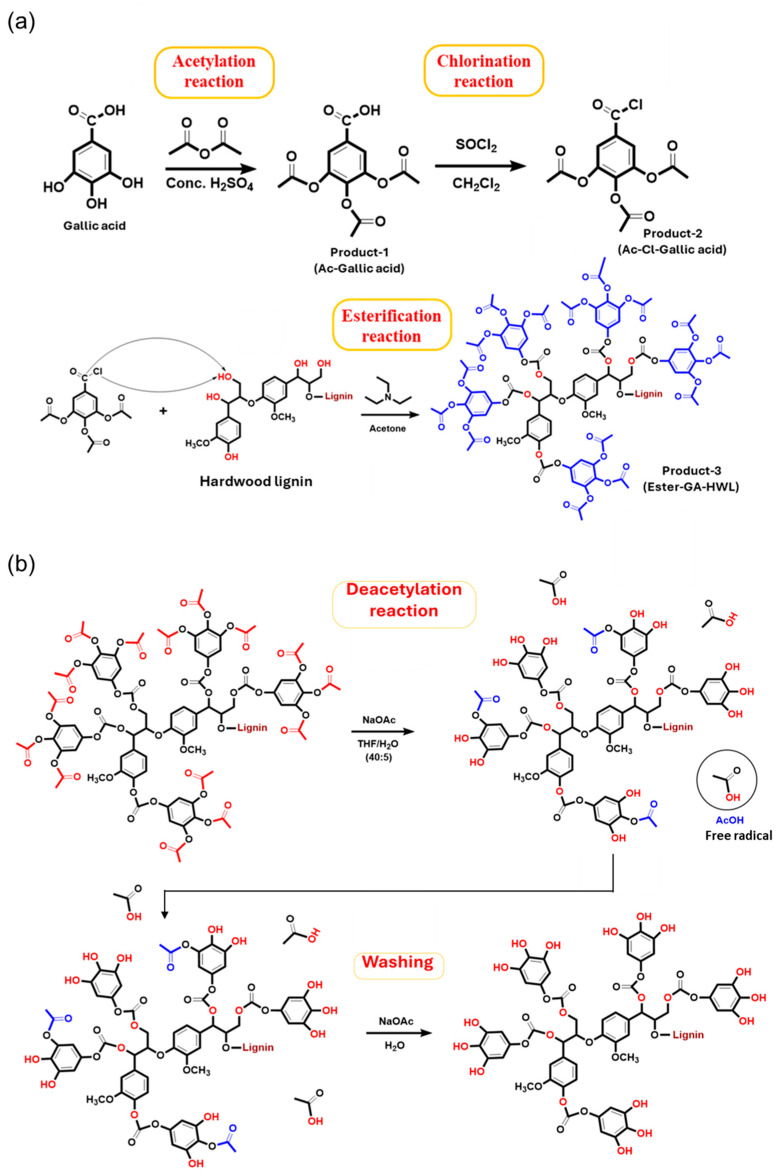
Schematic representation of the multi-step synthesis of gallic acid-grafted hardwood lignin (GA-graft-HWL) [41]. (**a**) Acetylation, chlorination, and esterification of gallic acid with hardwood lignin. (**b**) Deacetylation and washing to yield GA-graft-HWL. Ac-Gallic acid, acetylated-gallic acid; Ac-Cl-Gallic acid, acetylated-chlorinated-gallic acid; Ester-GA-HWL, esterified-gallic acid-hardwood lignin.

Esterification changes lignin structure by converting aliphatic and phenolic OH groups into ester bonds (–O–C(=O)–R). Typical routes include (i) chemical acylation using anhydrides or acyl chlorides and (ii) selective biocatalysis. A bioactivity-preserving example is the lipase-catalyzed esterification of the lignin-derived monolignol dihydroconiferyl alcohol (DCA) with fatty acids, in which the study emphasized selectivity for aliphatic OH groups to avoid phenolic masking. Product yields up to 97% were reported under solvent-assisted conditions for 24 h with two equivalents of fatty acid and under solvent-free conditions for 3 h with 10 equivalents [45]. Esterification reduces hydrogen bonding and polarity, increases hydrophobicity and compatibility with oils and polymers, and generally diminishes ionizable groups. If phenolic OH groups are esterified, phenolic accessibility and redox reactivity typically decrease; therefore, selective aliphatic esterification is used to maintain phenolic function while tuning solubility in hydrophobic matrices [45]. When phenolic OH groups are preserved, antioxidant capacity can remain high. In DPPH kinetics, the octanoate ester 4-(3-hydroxypropyl)-2-methoxyphenyl octanoate (DCA-C_8_) was slower initially but reached similar long-term stoichiometric quenching at 1.1 µM after 22 h compared with DCA, butylated hydroxytoluene (BHT), and butylated hydroxyanisole (BHA). Its 90 min IC_50_ was 12.0 µM (DCA 9.9 µM; BHA 10.4 µM; BHT 15.7 µM; and propyl gallate 3.5 µM) [45]. This supports esterification as a strategy to introduce lignin phenolics into hydrophobic application spaces, such as cosmetics, oils, and polymer matrices, without fully sacrificing radical scavenging (Figure 3). Antimicrobial and anti-inflammatory effects are less consistently quantified for simple esterification alone; mechanistically, greater hydrophobicity may enhance membrane association, but reduced aqueous solubility may limit bioavailability unless the material is formulated (e.g., as nanoparticles) [24,45].

### 4.2. Etherification

Etherification chemical structural change forms C–O–C linkages by O-alkylation or hydroxy-alkylation of lignin hydroxyls. A widely cited reagent class is alkyl oxides as an ethylene oxide or propylene oxide and epoxide chemistry epichlorohydrin, diglycidyl ethers [46]. A recent high-efficiency etherification determined and summarized phenolic-OH and aliphatic-OH etherification efficiencies around 98% and 96%, respectively, and noted enhanced solubility in common organic solvents chloroform, dilated cardiomyopathy (DCM), tetrahydrofuran (THF), and acetone [50]. Etherification physicochemical effects typically decrease OH acidity and hydrogen bonding, raising hydrophobicity and organic solubility for alkyl ethers; conversely, hydroxy alkylation can introduce new aliphatic OH and sometimes increase solubility in polar media. Net charge remains near neutral unless ionic ether substituents (e.g., carboxymethyl) are used. Phenolic accessibility can decrease dramatically if phenolic OH is converted. Chemical reactivity shifts toward ether structures and reduces phenolic redox participation [57,59]. Bioactivity consequences of etherification—the dominant antioxidant mechanism—relies on phenolic OH donation; extensive phenolic etherification is expected to reduce intrinsic radical scavenging per mass in aqueous assays; improvements may still be realized in nonpolar matrices through improved dispersion and retention of some phenolic sites [44,45]. Direct quantitative antimicrobial and anti-inflammatory data for etherified lignin are less standardized in open primary reports compared with cationized or phenolic enriched derivatives, so etherification is currently most viewed as enabling modification for processing, blending, and downstream functionalization rather than a primary bioactivity amplifier [46,50].

### 4.3. Phenolation

**Figure 4:**
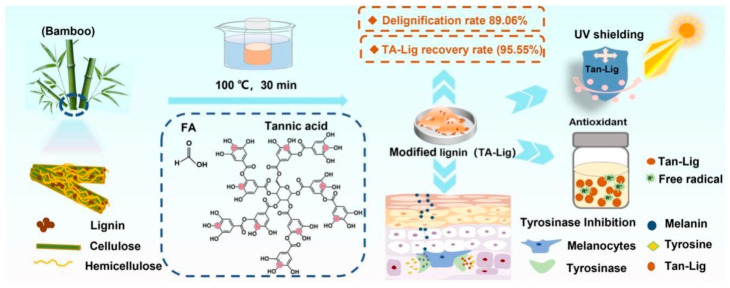
Schematic diagram of tannic acid-assisted formic acid pretreatment for the preparation of multifunctional lignin and its cosmetic application. Image adapted from Xu et al. [69] and reproduced with permission from Elsevier, ©2026.

Phenolation reaction increases the density of phenolic motifs by grafting phenolic species and generating additional phenolic termini onto lignin, often via oxidative coupling. A clear recent biological example uses tannic acid grafting for a polyphenol rich in phenolic OH onto lignin through laccase oxidation under high-intensity ultrasound, simultaneously driving phenolation and nanoparticle formation to phenolated lignin nanoparticles (PheLigNPs). Physicochemical effects of the phenolation generally increase phenolic OH content. Tannic acid grafting increases the phenolic density of lignin, which enhances its hydrophilicity and improves surface properties (Figure 4). As a result, this modification enhances the compatibility of lignin with water-based systems, making it more suitable for various applications. Net charge can become more negative at neutral/basic pH via phenolate formation, but pH-dependent. Oxidative coupling can also introduce crosslinks that alter M_w_ distribution and reactivity. Bioactivity-increased phenolic density can enhance antioxidant capacity and can also drive antimicrobial effects via (i) membrane interactions and (ii) oxidative stress induction in microbes. In PheLigNPs, reported MICs were 1.25 mg/mL^−1^ for *Staphylococcus aureus* and *Bacillus cereus*, and 2.5 mg/mL^−1^ for *Pseudomonas aeruginosa* and *Escherichia coli*; MICs of nonfunctional lignin nanoparticles (LNPs), bulk-phenolated lignin, and pristine lignin were described as under to 2 × higher, supporting synergy of phenolic enrichment and nanoscale presentation. Chemical modifications, such as phenolic grafting or esterification, can significantly alter lignin’s bioactivity. For instance, gallic acid grafting has been shown to increase the phenolic hydroxyl content, thereby enhancing antioxidant and antimicrobial activities. Such modifications not only affect the chemical reactivity of lignin but also influence its solubility, hydrophilicity, and dispersion, further impacting its biological interactions [27]. Mechanistically, bacteria in contact with PheLigNPs showed decreased metabolic activity and elevated ROS, consistent with a multi-hit antibacterial mode rather than a single enzyme target, which may reduce resistance propensity relative to antibiotics [68].

### 4.4. Amination

Amination modification chemical structural introduces C-N functionalities, typically as aminoalkyl substituents on the aromatic ring via the Mannich reaction (phenolic substrate + formaldehyde + amine). In an amine-functionalized kraft lignin study, the Mannich reaction was used to introduce C-N functionalities [49]. In the first step, formaldehyde reacts with a primary or secondary amine—in this case, diethylenetriamine (DETA)—to form an iminium ion intermediate. This intermediate then undergoes electrophilic substitution at the ortho or para position relative to the phenolic hydroxyl group on the lignin aromatic ring, generating stable C-N linkages and introducing primary and secondary amine functionalities onto the lignin backbone. Amination physicochemical effects from amines protonate in mildly acidic to physiological conditions, increasing net positive charge and often improving aqueous dispersion. The nitrogen appearance and strong N-H/C-N spectroscopic signatures, with reported nitrogen atomic percent, increase from 0.89 to 13.37% after amination. Amination can increase chemical reactivity (further quaternization, coupling, and crosslinking) and can increase redox-active, adjacent group effects by electron donation near phenolic OH, plausibly lowering O-H bond dissociation energies if the magnitude is system-specific [25]. Bioactivity consequences of antioxidant improvements are often reported at a functional level (e.g., radical scavenging role in polymer/rubber aging resistance) rather than only DPPH. The amine-functionalized lignin work explicitly attributes improved anti-aging performance to a radical scavenging effect, positioning amination as a route to replace conventional amine antioxidants with toxic transformation products. Aminated lignin nanoparticles have been reported to show enhanced antioxidant activity compared with unmodified lignin nanoparticles. However, antimicrobial outcomes are not consistently improved: in one study, aminated lignin nanoparticles showed reduced inhibition of *S. aureus* relative to unmodified nanoparticles. This was attributed to particle morphology changes caused by amination, which may hinder nanoparticle penetration through the bacterial cell wall [68]. Anti-inflammatory effects are typically indirect as ROS suppression unless coupled to specific anti-inflammatory motifs graft-copolymer hydrogels provide clearer evidence [70].

### 4.5. Sulfonation

Sulfonation introduces sulfonate groups (-SO_3_^−^) on aromatic units, forming lignosulfonates. Industrially, this occurs in sulfite pulping, giving randomly sulfonated phenylpropane polymers. A study on lignosulfonic acid sodium (LSAS) paper describes LSAS as randomly sulfonated lignin, water soluble, derived from sulfite pulping, and functioning as a non-saccharide heparin mimetic. Sulfonate groups impart strong anionic charge, high water solubility, and polyelectrolyte behavior. This often lowers hydrophobicity and increases interaction with proteins via combined anionic, hydrogen-bonding, and hydrophobic interactions. Chemically, sulfonation tends to shift lignin into biointerface-active space for enzyme binding and electrostatic interactions rather than purely phenolic redox chemistry. LSAS shows quantified multi-modal effects, including inhibition of inflammation-related proteases such as human neutrophil elastase, cathepsin G, and plasmin, with IC_50_ values spanning 0.73–212.5 µg/mL. It also shows chemical antioxidant activity with IC_50_ 44.1 µg/mL in an ABTS/hydrogen peroxide/metmyoglobin system, and cellular anti-inflammatory action by inhibiting TNF-α-induced NF-κB activation in HEK-293 reporter cells, with strong inhibition at 526 µg/mL. These data support sulfonation as one of the most evidence-backed routes to anti-inflammatory lignin-derived polymers, with the additional advantage of existing industrial-scale lignosulfonate production [49].

### 4.6. Carboxylation

Carboxylation reaction process introduces -COOH/-COO^−^ groups through oxidation, carboxyalkylation, or grafting of dicarboxylic acids. A mechanistically explicit recent example is maleic acid hydrotropic fractionation (MAHF), which reports that lignin is carboxylated by maleic acid at the γ-OH of the side chain during fractionation, changing both chemical composition and supramolecular behavior [67].

Carboxyl groups increase hydrophilicity and generate negative charge at neutral pH, improving colloidal stability. In MAHF-isolated acid hydrotropic lignin, nanoscale particles up to 100 nm and ζ-potential values lower than −20 mV were reported. These properties were attributed to abundant surface phenolic OH and carboxyl groups that stabilize dispersions by electrostatic repulsion [67]. Carboxylation also increases chemical reactivity for coupling, such as EDC/NHS amidation and esterification, and can tune the hydrophobicity/charge balance for biomedical carriers [67,71].

In the MAHF study, carboxylated lignin from wheat straw showed radical-scavenging rates up to 98% toward DPPH and 94% toward ABTS. The authors reported a positive correlation of scavenging with carboxyl content, phenolic hydroxyl content, and S-/G-unit ratio, arguing that carboxylation provides additional antioxidant activity alongside phenolic OH [67]. Antimicrobial and anti-inflammatory data for carboxylation are less standardized than those for sulfonation or quaternization. However, carboxylates provide a strong platform for downstream conjugation with phenolics, peptides, and drugs and for stable nanoparticle formation, which can translate to improved biological performance when paired with active modification [41,72].

### 4.7. Quaternization

The quaternization installs permanent quaternary ammonium groups (R^4^N^+^), commonly via chloromethylation followed by nucleophilic substitution with tertiary amines, producing cationic lignin salts with tunable alkyl chains. Furthermore, synthesized C_6_–C_18_ QAL from organosolv lignin (hardwood) is confirmed by spectroscopies and increased ζ-potential and tested antibacterial activity against clinical *K. pneumoniae* and methicillin-resistant *S. aureus*. Physicochemical effects of quaternization modification strongly increase positive charge around reported ζ-potential often under to +40 mV, improving dispersion, while hydrophobicity increases with longer alkyl chains for membrane insertion [68]. The same study discusses diffusion as an optimum in agar assays (C_12_–C_14_) consistent with cut-off effects from reduced solubility and diffusion at very long chains. Phenolic OH may remain but can become less decisive because membrane disruption dominates bactericidal action [68]. Quaternization is one of the highest potency antimicrobial modifications reported for lignin. Longer-chain QAL (C_14_–C_18_) achieved MBC as low as 0.012 mg/L against *K. pneumoniae* after 1 h and reached similar effect sizes for *S. aureus* by 24 h activity increased with alkyl chain length and correlated with ζ-potential for some lignin sources. Mechanistically, the cationic head group promotes electrostatic adhesion to negatively charged bacterial envelopes, while the hydrophobic tail disrupts membrane integrity, a well-established quaternary ammonium compound (QAC) paradigm [68]. Anti-inflammatory activity is not typically the primary target of quaternization instead; QALs are best positioned for coatings, packaging, and medical textiles where rapid bactericidal action is required to be balanced against careful cytotoxicity and environmental persistence assessment [24].

### 4.8. Graft Polymerization

Graft polymerization covalently attaches polymer chains onto lignin or graft-from, changing chemical structure. Bioactivity-relevant outcome, a lignin polyoxazoline system lignin, was tosylated to create a macroinitiator 2-methyl oxazoline that underwent ring opening polymerization from tosyl sites to form lignin-graft-polyoxazoline (lig-*g*-POZ). The copolymer was further modified by conjugation of a triazole moiety to reinforce antimicrobial and antibiofilm activity [72]. Also, lignin-grafted polylactic acid (lignin PLA) system was grafted into lignin via ring opening polymerization of lactide (DBU-catalyzed) following a dodecylation pretreatment, enabling electrospun PLLA/LP nanofibers with tunable lignin content and antioxidant performance. Grafting tunes’ physicochemical effects for solubility and charge indirectly by polymer choice polyoxazoline increases polar-solvent compatibility and yields smoother, smaller nanoparticles; in one report, dynamic light scattering (DLS) size decreased from 50 to 100 nm (sulfonated lignin) to 15–30 nm after polyoxazoline grafting [72]. In PLLA/LP nanofibers, grafting improves miscibility in the hydrophobic matrix. The authors note that antioxidant inhibition increased with lignin content and reached 90% inhibition after 24 h for most composite fibers, showing time-dependent accessibility effects in a solid scaffold [44].

Grafting can both shield phenolics (lower immediate reactivity) and improve dispersion (higher long-term effective activity) [72]. Furthermore, bioactivity of grafted systems provides some of the clearest multi-functional bioactivity evidence. The lig-*g*-POZ hydrogel was reported to down-regulate IL-1β expression and reduce iNOS and nitric oxide (NO) production in a pro-inflammatory macrophage model, with NF-κB protein expression also suppressed. Additionally, the system was designed as an anti-infective ointment as follows: after triazole conjugation, it demonstrated both antimicrobial and antibiofilm activity, and its wound-healing function was validated in vivo in an infected burn wound model [72]. For antioxidant biomaterials, PLLA/LP nanofibers showed strong radical inhibition that increased over time to 90% after 24 h for most and were positioned as protective scaffolds under oxidative stress in cartilage repair models.

Gallic acid (GA) and tannic acid conjugation typically forms ester or ether bonds between lignin aliphatic OH or carboxylated bonds and a bioactive phenolic molecule, increasing phenolic density. In our previous study, we grafted gallic acid onto hardwood lignin via a multi-step esterification sequence acetylation of GA phenolic OH, conversion to an acid chloride, esterification with hardwood lignin under base catalysis, and then deprotection to restore phenolic functionalities [41]. Phenolated lignin nanoparticles via tannic acid grafting with laccase and ultrasound represent a second polyphenol conjugation route [70]. Physicochemical effects of GA grafting substantially increase phenolic OH content and change hydroxyl distribution as follows: quantitative ^31^P NMR in the GA-grafted hardwood lignin report showed a fourfold increase in phenolic OH from 4.58 to 19.64 mmol/g and near-complete conversion of aliphatic OH to esters [41].

This shifts the balance toward higher hydrogen donating capacity as an antioxidant, potentially increases hydrophilicity of multiple OH, and increases chemical reactivity to more phenolic sites, altering ester content. Tannic acid conjugation similarly increases phenolic density and, when presented as nanoparticles, increases effective exposure [70]. The GA-grafted lignin report provides unusually strong quantitative antioxidant outcomes around 98.71% DPPH scavenging and complete ABTS neutralization, outperforming native hardwood lignin and approaching the efficacy of BHA in their comparisons as a positive control [41]. Tannic acid phenolated lignin nanoparticles showed enhanced antibacterial MIC 1.25–2.5 mg/mL^−1^ range across Gram-positive (Gram+) and Gram-negative (Gram-) examples versus nonfunctional NPs and bulk lignin, with ROS-mediated bacterial stress responses [70].

Anti-inflammatory effects are strongly supported when conjugation is paired with the lignin-graft-polyoxazoline system using sulfonated lignin as a starting material and further triazole conjugation-inhibited lipopolysaccharide (LPS)-stimulated induced inflammatory cytokine signaling pathway suppression of TNF-α/IL-1β/IL-6 proteins inhibitions and NF-κB activity while also acting as an anti-infective wound material [72].

### 4.9. Nanoparticle Formation

Nanoparticle chemical structural formation is not necessarily a new covalent bond strategy; rather it reorganizes lignin into nanoscale particles often via ultrasonication, solvent shifting, or precipitation. In an “Eco-friendly” approach for textiles, ultrasound reduced average particle diameters from 648 to 780 nm to 143–151 nm and lowered PDI; nanoparticles carried negative ζ-potential −23.8 to −25.8 mV, indicating stable dispersions in water [24].

Nanoparticle (NP) formation is frequently combined with chemical functionalization (phenolation, amination, and metal-binding), amplifying effects [24,70]. Nanoparticulate lignin increases surface area and exposes aromatic and phenolic groups at the interface; ζ-potential and surface chemistry control aggregation. The textile study explicitly linked negative ζ-potential to colloidal stability (double-layer repulsion). Phenolic accessibility is often improved, especially for additively or surface-functionalized NPs [24,70]. Biological activity of nanoparticle formation can improve both antioxidant and antimicrobial performance. In the textile-focused study, the most active nano-lignin sample reported DPPH IC_50_ to 10.38 µg/mL; inhibition zones against bacteria increased relative to bulk lignin, with reported bacterial inhibition zone ranges around 11.3–19.5 mm for nano-lignin versus from 9.3 mm to 13.7 mm for bulk lignin in their assay set [24]. Moreover, mechanistic antimicrobial evidence comes from phenolated lignin nanoparticles (a combined phenolation + nano strategy), where ROS elevation and reduced metabolic activity were observed in bacteria upon NP contact, consistent with oxidative damage with membrane disruption synergy [70]. Anti-inflammatory effects of lignin nanoparticles alone are less consistently quantified than antioxidant and antimicrobial outcomes. However, nanoparticle-enabled hydrogels and graft systems provide direct cytokine to NF-κB protein evidence [72].

### 4.10. Comparative Evaluation of Modification Methods

Lignin bioactivity modulation is best considered as selecting one of three dominant levers, then engineering around its tradeoffs. First lever is phenolic density and accessibility, targeted by phenolation and phenolic conjugation. GA-grafting provides a high confidence route to very high DPPH and ABTS antioxidant radical scavenging because it increases phenolic OH substantially while retaining the lignin backbone [41]. Phenolated lignin nanoparticle tannic acid-grafting demonstrates that phenolic enrichment can also enhance antimicrobial potency when coupled to nanoscale presentation and oxidative stress induction [70]. These are promising for food application antioxidants and packaging and for wound care surfaces where antioxidant and antimicrobial, assuming purification and leachable, are controlled. A second lever is net charge and membrane protein interactions, targeted by sulfonation and carboxylation (anionic) and amination and quaternization (cationic). Sulfonated lignosulfonates are compelling for anti-inflammatory and enzymatic modulation because they function as polymeric heparin mimetics: LSAS combines protease inhibition (IC_50_ down to sub-µg/mL for one target class), antioxidant activity (IC_50_ 44 µg/mL), and cellular NF-κB suppression at higher concentrations [49]. Carboxylation via maleic acid hydrotropic fractionation offers a scalable extraction with functionalization route; it generates anionic, colloidally stable lignin with high radical scavenging and clear structure–activity correlations with carboxyl and phenolic OH [67]. For antimicrobial applications that demand high potency, quaternization is dominant QALs that achieved bactericidal performance at extremely low concentration levels with tunable chain length structure activity, but this route may face stricter toxicology and green-chemistry scrutiny due to chloromethylation and quaternary ammonium environmental concerns [68]. Third lever is supramolecular presentation, targeted by nanoparticle formation and graft polymerization (Table 3). Nano-lignin can improve both antioxidant and antimicrobial activity, likely by increasing exposed functional groups and microbial contact. A study reported DPPH IC_50_ to 10.38 µg/mL and increased inhibition zones after ultrasound-mediated nanoparticle formation [24]. Graft copolymers and hydrogels are especially promising for biomedical contexts because they allow simultaneous tuning of biocompatibility, moisture handling, delivery, and signaling modulation. The lignin-*g*-polyoxazoline triazole platform explicitly demonstrated anti-inflammatory outcomes via iNOS, NO and NF-κBassociated assays alongside antimicrobial design goals [72].

## 5. Antioxidant and Antimicrobial Activities of Native and Modified Lignin

### 5.1. Antioxidant Activity

Lignin’s radical-scavenging capacity arises from the same phenolic architecture discussed in the preceding structural sections, yet the translation from chemical reactivity to measured antioxidant performance is mediated by a chain of confounding variables that are rarely controlled simultaneously. Protection factor (PF) and thiobarbituric acid reactive substance (TBAR) assays provide oxidative-stability endpoints integrated over extended time under realistic lipid oxidation conditions. Importantly, lignin samples that rank highly in DPPH are not guaranteed to maintain that ranking in PF or TBARs, because the kinetics of hydrogen-atom transfer in a lipid phase differ fundamentally from solution-phase radical quenching [50]. This catechol advantage is absent in most commercial technical lignins, whose extraction conditions selectively oxidize or condense catechol units, a key reason why native technical lignin typically performs below its theoretical phenolic maximum [55].

Choice of assay profoundly shapes the apparent ranking of lignin samples and must be considered critically. The DPPH and ABTS assays operate in organic or mixed-solvent systems where lignin is more readily dissolved, systematically overestimating activity relative to physiologically or food-relevant aqueous environments. Ferric reducing antioxidant power (FRAP) and reducing-power assays measure the capacity to donate electrons to ferric iron, an endpoint directly relevant to metal chelation in food matrices where iron-driven lipid peroxidation is the primary spoilage pathway. PF and TBARs provide oxidative-stability endpoints that integrate activity over extended time under realistic lipid oxidation conditions and lignin samples that rank highly in DPPH are not guaranteed to maintain that ranking in PF or TBARs, because the kinetics of HAT donation in a lipid phase differ fundamentally from solution-phase radical quenching [57]. The near-universal reliance on DPPH in published lignin work therefore skews the literature toward an overly optimistic view of lignin’s practical antioxidant utility in aqueous food or biological systems.

Extraction severity is a particularly underappreciated but experimentally documented determinant of antioxidant activity that is independent of molecular weight and phenolic content alone. Organosolv lignin fractions isolated under progressively harsher ethanol–water conditions from hybrid poplar demonstrated a monotonic decline in radical scavenging capacity as extraction intensity increased, even when phenolic hydroxyl content remained nominally constant across fractions, implicating condensation-driven steric burial of phenolic sites rather than simple loss of -OH groups as the mechanistic bottleneck [71]. This finding carries the following direct practical implication: reporting phenolic -OH content from ^31^P NMR without simultaneously characterizing the degree of condensation (β–5, 5–5′ units by HSQC) provides an incomplete and potentially misleading picture of antioxidant potential [55,71]. When lignin is processed into nanoparticles without chemical functionalization, antioxidant gains are realized primarily through improved surface exposure of existing phenolic groups rather than new chemical reactivity. Acid-isolated high-yield lignin nanoparticles (LNPs) showed measurably higher DPPH and FRAP values relative to their bulk precursor, and the improvement correlated with reduced particle size and increased specific surface area rather than with changes in phenolic OH content per gram [60]. However, the same nanoparticle stabilization that improves activity in dilute dispersions can be lost if LNPs aggregate under the ionic-strength conditions of food matrices or biological fluids—a stability issue that nanoparticle zeta potential alone does not fully predict, because bridging flocculation by divalent cations can collapse dispersions even at nominally repulsive surface charge values [56].

Hydrophobic modification presents a distinct antioxidant design challenge: increasing compatibility with polymer matrices and oils while retaining radical scavenging function. Microwave-assisted hydrophobization of kraft lignin allowed homogeneous dispersion in polylactic acid filaments; radical inhibition was maintained over extended incubation periods, positioning the material as a processing stabilizer against thermo-oxidative degradation during melt extrusion [44]. This contrasts with the aqueous assay results discussed above and highlights that the most useful antioxidant framing for lignin is application-specific: high DPPH activity is necessary but not sufficient for antioxidant performance in a solid polymer matrix, where diffusion of the radical target to the phenolic site, not electron transfer thermodynamics, is rate-limiting [69].

A persistent contradiction in the literature concerns the relationship between S/G-units and antioxidant strength. The intuitive expectation that S-rich lignin with doubly methoxylated phenoxyl radicals should be more stable and hence better antioxidants are not consistently confirmed experimentally. Comparisons across lignin types often show G-unit-rich softwood lignin performing on par with or above S-unit-rich hardwood lignin in DPPH assays, because higher condensation in softwood creates more C-C crosslinks that reduce solubility but also enrich the isolated fraction in lower-M_w_, more soluble components with higher phenolic density [55,57]. This underscores that S-/G-unit ratio functions as a proxy for multiple co-varying structural parameters not as an independent, mechanistically interpretable predictor, and its use as a standalone structure–activity descriptor in lignin antioxidant research is scientifically unsatisfactory [50,71].

### 5.2. Antibacterial Activity

The broad pattern across the antibacterial lignin literature is that Gram+ species, such as *Staphylococcus aureus* and *Bacillus* spp. are more consistently susceptible than Gram- species at equivalent lignin concentrations [57]. This is mechanistically expected: the outer membrane of Gram- bacteria constitutes an additional permeability barrier that limits access of large, hydrophobic lignin aggregates to the inner membrane target. Yet this pattern is frequently obscured in published inhibition zone studies because disk diffusion results are dominated by diffusion coefficients through agar which are inversely related to M_w_ rather than by intrinsic bactericidal potency. Native kraft and organosolv lignin reported to produce inhibition zones against Gram- organisms in disk diffusion assays should be treated with particular skepticism unless confirmed by MIC determination in broth, with appropriate turbidity controls [8,57].

Nanoparticle engineering can partially overcome the Gram- outer-membrane barrier by reducing particle size to the range where electrostatic or hydrophobic interactions with lipopolysaccharide become feasible. Silver-infused lignin core nanoparticles demonstrate this principle: sub-stoichiometric silver loading on a lignin scaffold achieved bactericidal efficacy while reducing total silver content approximately tenfold relative to equivalent ionic silver treatments, with the lignin matrix functioning as a spatial delivery platform that sustains local ion release at the bacterial surface rather than passively diluting silver into the bulk medium [66]. This system is notable because it uses lignin’s adsorptive and colloidal properties to amplify the activity of an exogenous biocidal agent, rather than relying exclusively on lignin’s intrinsic phenolic chemistry, a design principle with direct relevance to food-contact antimicrobial coatings where low-toxicity, low-migration formulations are regulatory requirements. Antifungal activity of lignin and lignin nanoparticles has been reported but is markedly less characterized than antibacterial effects, and the mechanistic basis is poorly established. The available evidence suggests that low M_w_, high phenolic fractions typically obtained from organosolv or enzymatic hydrolysis are the most active, consistent with membrane disruption via phenolic intercalation rather than surface charge interactions [57,71]. High M_w_ lignosulfonates, despite their excellent water solubility, show minimal antifungal activity, which is mechanistically coherent given that their activity profile is oriented toward protein polyelectrolyte interactions rather than membrane perturbation. The absence of standardized antifungal MIC protocols analogous to clinical and laboratory standard institute (CLSI) broth microdilution makes cross-study comparison even more difficult than for antibacterial assays.

Several methodological problems substantially weaken part of the antimicrobial literature. First, nutrient-rich broth media contain divalent cations (Ca^2+^, Mg^2+^) that bridge negatively charged lignin colloids, causing aggregation and sedimentation and reducing the effective contact concentration, an effect that is essentially never controlled in published work [8]. Second, the pH of the growth medium shifts lignin surface charge and solubility, alkaline conditions that deprotonate phenolic OH groups improve dispersion but may simultaneously reduce membrane active hydrophobicity. Third, many studies report a single-concentration inhibition result (e.g., percent growth inhibition at 1 mg/mL) rather than a full concentration response MIC or MBC profile, preventing any assessment of potency versus efficacy. Taken together, claims of antimicrobial activity based solely on disk diffusion zone diameters for native or lignosulfonate lignin represent weak evidence; the strongest mechanistically supported claims involve functionalized nanoparticle systems tested by broth microdilution with confirmatory metabolic activity or membrane integrity assays [66].

### 5.3. Anti-Inflammatory and Health-Related Biological Activities of Native and Modified Lignin

The anti-inflammatory response of lignin operates through at least two mechanistically distinct modes, indirect attenuation through ROS scavenging which reduces the oxidative substrate that activates NF-κB and direct modulation of cytokine signaling cascades and inflammatory protein expression, which requires specific structural features not present in most unmodified technical lignins [49,72].

**Figure 5:**
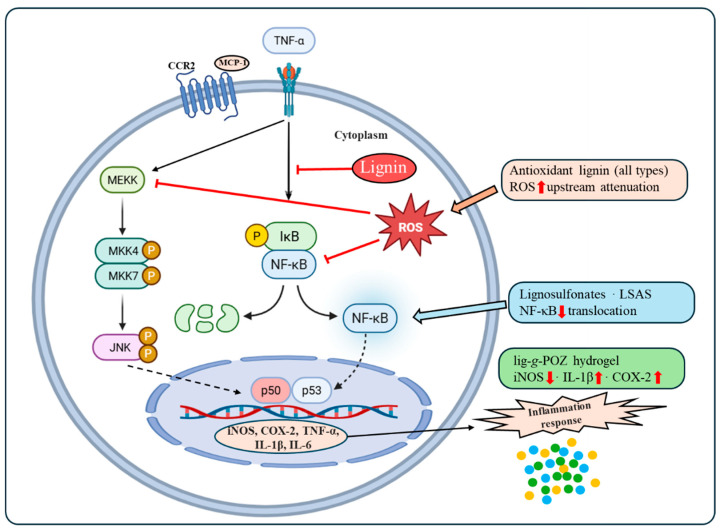
Pro-inflammatory cytokine signaling pathway of modified lignin effects. Black solid arrows indicate activation or pathway progression, red lines with blunt ends indicate inhibition or suppression, and dashed arrows indicate translocation into the nucleus.

Distinguishing between these modes is important because ROS-mediated effects are a predictable extension of antioxidant activity and can be recovered with any sufficiently active radical scavenging fraction. By contrast, direct cytokine pathway modulation represents higher biological specificity and greater therapeutic relevance (Figure 5).

Evidence for direct inflammatory pathway modulation in lignin-related phenolic systems includes the suppression of TNF-α-induced vascular cell adhesion molecule-1 (VCAM-1) expression in human umbilical vein endothelial cells (HUVECs) by phenolic-rich plant extracts with lignin-adjacent polyphenol composition [36]. VCAM-1 induction is a key upstream event in vascular inflammatory recruitment, regulated through NF-κB activation, and its suppression by polyphenolic structures at the endothelial cell surface points to a membrane proximal inhibitory mechanism potentially involving interference with TNF-α receptor signaling or IκB kinase complex activity that is consistent with, but not limited to, lignin phenolic scaffold [42,59]. While this study does not use isolated lignin, the structural parallels to lignin-derived phenolics motivate targeted mechanistic investigation in well-characterized lignin fractions.

Alkaline lignin fractions from non-standard plant sources have been shown to exhibit immunomodulatory behavior alongside antimicrobial and cytotoxic properties. Alkaline lignin extracted from *Morinda citrifolia* leaves demonstrated inhibitory activity against tumor cell proliferation in vitro and modulation of immune macrophage cell function, effects attributed to the combined action of phenolic OH groups on pro-inflammatory cytokine-relevant transcription factors and the capacity of high M_w_ polyphenolic polymers to engage pattern recognition receptors on macrophage surfaces [42]. Although the mechanistic resolution in this study is limited, cytokine quantification and NF-κB reporter assays were not reported; the observation that anti-inflammatory and antitumor effects co-segregate with the polyphenolic lignin fraction, rather than with co-isolated alkaloids or flavonoids, provides a basis for more targeted fractionation and pathway-specific investigation [42].

At the protein expression level, the most relevant inflammatory targets for lignin-based materials are iNOS, COX-2, NF-κB, IL-1β, IL-6, and TNF-α cytokine triad. iNOS upregulation drives sustained nitric oxide production in activated macrophages and is a direct amplifier of oxidative tissue damage; COX-2 induction governs prostaglandin synthesis and sustains the inflammatory pain and edema response (Figure 5) [83]. Suppression of both enzymes, alongside NF-κB nuclear translocation, is the mechanistic benchmark for anti-inflammatory materials [49]. The current evidence suggests that sulfonated and graft-copolymer lignin derivatives are more capable of achieving this multi-target suppression than native or simple esterified lignin, because polyanionic character enables competitive interaction with the heparin-binding interfaces of inflammatory mediators, while graft-copolymer architectures allow co-presentation of anti-inflammatory and antimicrobial motifs in a single biointerface [49,72].

Broader health-related activities reported for lignin derivatives include antiviral effects, anticoagulant effects, and inhibition of α-glucosidase relevant to glycemic control, all of which are documented in the reviewed literature [58]. Lignosulfonates have been shown to interfere with HIV and influenza surface-protein binding through steric and electrostatic competition with heparan sulfate receptors. These diverse activities converge on the same structural feature; polyanionic, phenolic polymers can engage multiple protein binding sites non-specifically. Whether this breadth of in vitro activity translates to useful in vivo function depends critically on bioavailability, which remains essentially uncharacterized for most technical lignin fractions in oral or systemic administration scenarios. Biodegradable lignin nanoparticles have shown enhanced antiproliferative effects in cancer cell lines relative to solubilized lignin, attributed to nanoparticle-mediated intracellular ROS generation and mitochondria-driven apoptosis induction. This suggests that formulation format, and not just chemical structure, governs the biological outcome even for identical lignin compositions [53]. This observation reinforces the conclusion that the biomedical relevance of lignin derivatives cannot be assessed from chemical characterization and in vitro assay data alone; delivery form, dose kinetics, and tissue-specific exposure are co-equal determinants of the outcome [83].

## 6. Functional Food Properties

Modified lignin is being explored as multifunctional food additive. In packaging and coatings, it can enhance barrier, UV-shielding, antioxidant and antimicrobial functions; as a Pickering emulsifier, it can stabilize bioactive oils; and as a feed supplement, it may act as dietary fiber or a prebiotic. These uses exploit lignin’s phenolic chemistry, but face challenges related to color, migration, cost, and regulatory data. They also require tailored modifications, including nanoparticles, grafting, and enzymatic treatments.

### 6.1. Packaging Food and Edible Coatings

Lignin-modified biopolymers are extensively studied for active packaging and edible coatings. Incorporating nanoparticles and grafted lignin copolymers into starch, chitosan, PLA, poly(vinyl alcohol) or gelatin films can markedly improve functionality. Lignin’s high phenolic content imparts strong antioxidant and UV-blocking activity, while its aromatic structure enhances water and oxygen barrier properties and hydrophobicity [83,84]. For instance, adding 1–3 wt% lignin nanoparticles to starch or pectin films boosted tensile strength by up to 164% and contact angle by 48–56%, while reducing water vapor permeability by 25% [85]. Chitosan lignin composites likewise gained radical-scavenging activity with only slight loss of film toughness [84]. Active agents (e.g., essential oils) can be co-delivered via lignin-stabilized Pickering emulsions. One study embedded a 3% lignin–clove–oil emulsion in starch films, yielding 40% tensile strength, a water contact angle of 91.17°, and DPPH and ABTS radical scavenging activities of 87–94%, and antibacterial rates above 79% against *E. coli* and *S. aureus* [14]. In practice, such films delayed tomato spoilage (80% decay reduction) while matching plastic wrap moisture control [14,86]. One limitation is that lignin’s brown color limits reduce transparency, so formulations must balance UV-protection with appearance [83]. High loadings above 5% often cause nanoparticle aggregation and property loss. Importantly, all food-contact coatings require demonstration of safety. Although lignin itself is generally regarded as inert, nanoparticulate lignin lacks toxicological data, and potential migration of phenolics into food is largely untested. No definitive regulatory approvals exist for lignin in food packaging, so full compliance studies are still needed [85,87].

### 6.2. Emulsion Stabilization and Additives

Lignin’s amphiphilic, polyaromatic structure makes it a natural emulsifier. LNPs readily stabilize oil-in-water Pickering emulsions, enabling encapsulation of bioactive oils or flavors [88].

For instance, LNPs can entrap essential oils in films or beverage emulsions, adding antimicrobial and antioxidant effects without synthetic surfactants [14]. Enzymatically or chemically modified lignin (e.g., phenolated lignin) can further boost antimicrobial potency; studies report up to 99% inhibition of *E. coli* and *S. aureus* with such grafted lignin. Emulsions stabilized by LNPs can show high stability indices and controlled release, although exact metrics vary by system [89]. In one coating film, the LNP–Pickering emulsion delivered sustained release of clove oil over several weeks. One limitation is that formulating stable food emulsions with lignin may require control of pH and ionic strength. As with films, any leaching of lignin bound compounds into foods must be evaluated for taste or toxicity [90].

### 6.3. Animal Feed Supplements

Unmodified lignin is largely indigestible, so as a fiber it has limited nutritional value and can be antinutritional at high levels. However, some studies suggest benefits at low inclusion rates. Purified lignin from kraft or sulfite pulping contains low phenolic compounds that may exhibit prebiotic and antimicrobial effects in the gut [91].

For instance, Alcell (kraft) lignin at 1–4% of the diet improved growth in poultry and calves. This lignin increased beneficial gut bacteria (e.g., Bifidobacterium) and improved intestinal morphology. A recent trial with 1% sugarcane bagasse lignin in chicken feed found better pellet durability, reduced microbial spoilage, and higher Bifidobacterium counts in mash diets [89].

Limitation above 5% of the diet, purified lignin often yields no growth benefit and can accelerate gut transit or impair nutrient absorption. Monogastric animals, such as pigs and poultry, cannot digest lignin bonds, so high levels dilute dietary energy of protein. Some lignosulfonate lignin is already authorized (e.g., calcium lignosulfonate up to 1% feed), reflecting safety at low doses. Still, formal approval of new lignin feed additives requires hazard data [92].

### 6.4. Safety, Regulatory and Implementation

No exotic hazards are known for lignin beyond its phenolic nature. Bulk lignin is not genotoxic, but nanosized forms and residual pulping chemicals must be evaluated. Key practical challenges include: (i) migration: data on lignin or nanoparticle leachates in food are scarce, impeding EFSA/FDA approval; (ii) sensory quality: lignin imparts brown color and possible astringency, so additives must not alter taste or appearance; (iii) cost: lignin is cheap byproduct, but its valorization requires extra steps, such as nanoparticle formation and grafting. High-tech methods (e.g., polymer grafting via ROP) add processing cost, and current yields may not compete with cheap additives such as TiO_2_ and ZnO [93]. (iv) Compatibility: lignin often needs dispersion aids or covalent linking (e.g., forming lignin polyester copolymers) to mix well with target polymers. For edible films, simple mixing or mild enzyme crosslinking may suffice, whereas packaging films may require reactive extrusion or compatibilizers to avoid phase separation [94].

## 7. Conclusions and Future Perspectives

In conclusion, this review shows that native lignin has the best intrinsic biological potential because its structure is less degraded and its phenolic functionality is better preserved. However, for real applications, technical lignin is more practical. Among technical lignins, organosolv and soda lignin appear most promising because they are sulfur-free, less condensed, and more reactive than kraft lignin, making them better starting materials for bioactive and functional food-related applications. At the monomer level, catechol type lignin-derived structures show particularly strong antioxidant potential because the *ortho*-dihydroxy arrangement stabilizes radicals effectively. For direct application, vanillin remains the most attractive monomer because it combines recognized food relevance with antioxidant, antimicrobial, and anti-inflammation effects. This makes vanillin one of the clearest bridges between lignin modification and human health-related applications.

Among modification routes, simple and scalable approaches such as esterification, carboxylation, and phenolic grafting are more realistic for industry than complex multi-step polymer grafting. Still, the best bioactivity results were obtained when lignin was both chemically enriched and structurally optimized. Gallic acid grafting achieved near-complete ABTS neutralization and 98.7% DPPH scavenging activity, together with a fourfold increase in phenolic hydroxyl content.

Maleic acid-based carboxylation also reached about 98% DPPH and 94% ABTS scavenging activity. For antimicrobial activity, phenolated lignin nanoparticles showed MIC values of 1.25 mg/mL against *S. aureus* and *B. cereus*, while nano-lignin reduced DPPH IC_50_ to 10.38 µg/mL and increased bacterial inhibition zones to about 11.3–19.5 mm. For anti-inflammatory activity, sulfonated lignosulfonates showed strong protease inhibition with IC_50_ values as low as 0.73 µg/mL and antioxidant IC_50_ around 44.1 µg/mL, together with NF-κB suppression. Overall, phenolic grafting, especially with gallic acid and tannic acid, combined with nanoparticle engineering appears to be the most promising low-cost future route for food and human health applications. This strategy is simple, effective, and scalable, but still requires deeper toxicity, migration, and structure–activity validation.

## Figures and Tables

**Table 1:** Lignin-derived phenolic monomers: chemical structures, bioactivities, and applications.

Compound (Unit)	Chemical Structure	Bioactivities	Applications	References
*p*-Coumaric acid (H-type)	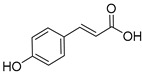	Antioxidant; anti-inflammatory; antimicrobial	Natural antioxidant (foods, cosmetics); antifungal/stabilizer	[20]
Caffeic acid (G-type)	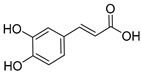	Strong antioxidant, anti-inflammatory, anticancer (general phenolics)	Food ingredient, nutraceuticals	[20]
Ferulic acid (G-type)	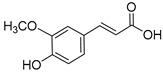	Potent antioxidant, antimicrobial, anti-inflammatory	Food/pharma additive, skin care	[21]
Sinapic acid (S-type)	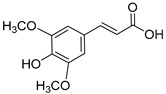	Very strong antioxidant; antimicrobial; anti-inflammatory, anticancer	Food antioxidant, anti-inflammatory agent	[22]
Vanillin (G-type)	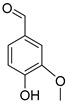	Antibacterial (MIC 1–2.5 mg/mL), antioxidant, anti-inflammatory	Flavoring; preservative; pharma intermediate	[23]
Syringaldehyde (S-type)	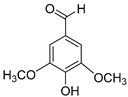	Antioxidant, antimicrobial (similar to vanillin)	Flavor, fragrance, polymer precursor	[24]
Flavonoids	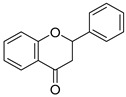	Antioxidant, anti-inflammatory, antimicrobial	Nutraceuticals, cosmetics	[25]

**Table 3:** Overview of chemical, enzymatic, and physical methods to modify lignin, and biological properties.

Method	Mechanism	Bioactivity Effect	Scale/Cost	Functional Food Properties	References
Sulfonation (lignosulfonates)	Introduce SO_3_^−^ groups (via sulfite pulping)	Strong antimicrobial (surfactant effect); modest antioxidant	High (commercial pulp byproduct) low cost	Water-soluble emulsifier; potential prebiotic fiber; antimicrobial preservative in food coatings	[62]
Carboxylation (oxidation or maleic acid)	Add –COOH to side-chains or phenols	Greatly enhanced antioxidant slight increase in acidity	Medium (requires oxidants or maleic acid)	Mineral chelation (Fe^2+^, Ca^2+^); enhanced antioxidant activity in food matrices; improved emulsion stability	[73,74]
Acetylation	Convert –OH to –OAc (using acetic anhydride)	Increases hydrophobicity; modest effect on antioxidant	Low to medium (uses acetic anhydride or acid chloride)	Improved emulsification of fat-based systems; hydrophobic encapsulant for flavor/aroma compounds	[75]
Polymer grafting (e.g., PLA, PEG, styrene)	Covalent attachment of polymers (via ATRP/RAFT/ROP)	Improve mechanical/ solubility and functionalities	Low yield, high cost (complex catalysts)	Bioactive encapsulation and controlled release; edible film/packaging; texture-modifying agent	[76]
Click chemistry (CuAAC, SPAAC, Diels–Alder)	Link azide/alkyne or furan groups to lignin	Precise addition of bioactives or functionality	Low scale, costly reagents (e.g., Cu catalyst)	Precise conjugation of nutraceuticals (vitamins, polyphenols); targeted antioxidant/antimicrobial delivery	[77,78]
Enzymatic (laccase, etc.)	Oxidative coupling or grafting of phenolics	Mild, green; increase crosslinking and phenolic content	Low rate, enzyme costs high	Clean-label crosslinker for gels/hydrogels; texture enhancer; enriched phenolic content for antioxidant fortification	[79]
Nanoformulation (LNPs)	Precipitation into nanoparticles	Strongly boosts antioxidant and antimicrobial activity	Low yield (1–5%), requires solvents or equipment	Enhanced bioavailability of lipophilic nutrients; controlled gut release; nano-encapsulation of vitamins/polyphenols	[57,80,81]
Thermal treatment	Heat degradation/ condensation	Increase phenolics by cleaving bonds, form quinones (antioxidant)	High temp—energy cost	Natural processing compatibility; increased free phenolic bioavailability; antioxidant fortification via quinone formation	[82]

## Data Availability

No new data were created or analyzed in this study. Data sharing is not applicable to this article.

## Abbreviations

The following abbreviations are used in this manuscript:

M_w_	Molecular weight
H-unit	*p*-Hydroxyphenyl unit
G-unit	Guaiacyl unit
S-unit	Syringyl unit
PLA	Polylactic acid
HCT	Hydroxycinnamoyl-CoA shikimate/quinate hydroxycinnamoyltransferase
C3H	p-coumarate 3-hydroxylase
CCoAOMT	Caffeoyl-CoA O-methyltransferase
F5H	Ferulate 5-hydroxylase
COMT	Caffeic acid O-methyltransferase
CCR	Cinnamoyl-CoA reductase
CAD	Cinnamyl alcohol dehydrogenase
C-lignin	Catechyl lignin
DES	Deep eutectic solvents
SAR	Systemic acquired resistance
PDI	Polydispersity index
DPPH	2,2-diphenyl-1-picrylhydrazyl
MIC	Minimum inhibitory concentration
MBC	Minimum bactericidal concentration
NF-κB	Nuclear factor kappa-B
NMR	Nuclear magnetic resonance
HSQC	Heteronuclear single quantum coherence
SEC	Size-exclusion chromatography
EC_50_	Half-maximal effective concentration
HAT	H-atom transfer
SET	Single-electron transfer
ROS	Reactive oxygen species
ABTS	2,2′-azino-bis-(3-ethylbenzothiazoline-6-sulfonic acid)
QAL	Quaternary ammonium lignin
TNF-α	Tumor necrosis factor-α
iNOS	Inflammatory mediators
DCA	Dihydroconiferyl alcohol
DCA-C_8_	Octanoate ester 4-(3-hydroxypropyl)-2-methoxyphenyl octanoate
BHT	Butylated hydroxytoluene
BHA	Butylated hydroxyanisol
IC_50_	Half-maximal inhibitory concentration
DCM	Dilated cardiomyopathy
THF	Tetrahydrofuran
Ac-Gallic acid	Acetylated-gallic acid
Ac-Cl-Gallic acid	Acetylated-chlorinated-gallic acid
Ester-GA-HWL	Esterified-gallic acid-hardwood lignin
PheLigNPs	Phenolated lignin nanoparticles
LNPs	Lignin nanoparticles
DETA	Diethylenetriamine
LSAS	Lignosulfonic acid sodium
MAHF	Maleic acid hydrotropic fractionation
EDC	1-Ethyl-3-(3-dimethylaminopropyl)carbodiimide
NHS	N-hydroxysuccinimide
QAC	Quaternary ammonium compound
lig-*g*-POZ	Lignin-graft-polyoxazoline
lignin PLA	lignin-grafted polylactic acid
PLLA	Poly(L-lactic acid)
LP	Lignin-PLA
DLS	Dynamic light scattering
GA	Gallic acid
Gram+	Gram-positive strain
Gram-	Gram-negative strain
LPS	Lipopolysaccharide
NP	Nanoparticles
PF	Protection factor
TBARs	Thiobarbituric acid reactive substances
FRAP	Ferric reducing antioxidant power
CLSI	Clinical and laboratory standard institute
VCAM-1	Vascular cell adhesion molecule-1
HUVECs	Human umbilical vein endothelial cells
